# Revealing the Reaction Pathway of Anodic Hydrogen Evolution at Magnesium Surfaces in Aqueous Electrolytes

**DOI:** 10.1021/jacs.4c10086

**Published:** 2024-10-25

**Authors:** Florian Deißenbeck, Sudarsan Surendralal, Mira Todorova, Stefan Wippermann, Jörg Neugebauer

**Affiliations:** † Max-Planck-Institut für Eisenforschung GmbH, Max-Planck-Straße 1, Düsseldorf 40237, Germany; ‡ Philipps-Universität Marburg, Renthof 5, Marburg 35032, Germany

## Abstract

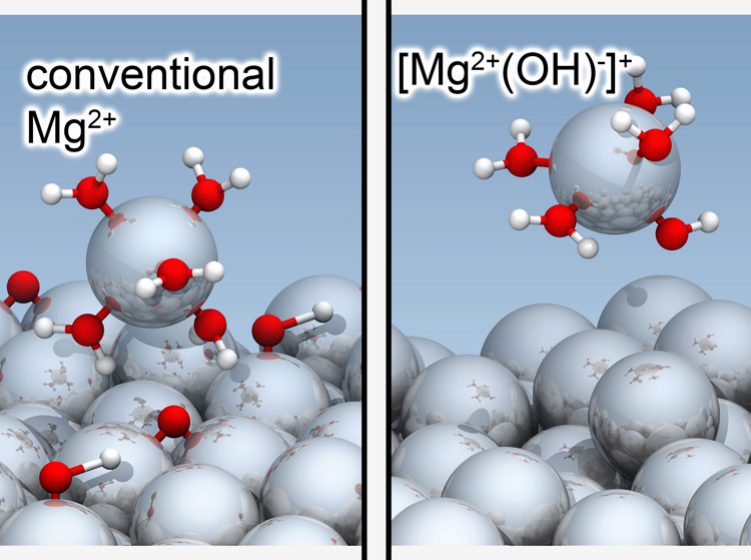

Aqueous metal corrosion is a major economic concern in modern society. A phenomenon that has puzzled generations of scientists in this field is the so-called anomalous hydrogen evolution: the violent dissolution of magnesium under electron-deficient (anodic) conditions, accompanied by strong hydrogen evolution and a key mechanism hampering Mg technology. Experimental studies have indicated the presence of univalent Mg^+^ in solution, but these findings have been largely ignored because they defy our common chemical understanding and evaded direct experimental observation. Using recent advances in the *ab initio* description of solid–liquid electrochemical interfaces under controlled potential conditions, we describe the full reaction path of Mg atom dissolution from a kinked Mg surface under anodic conditions. Our study reveals the formation of a solvated [Mg^2+^(OH)^−^]^+^ ion complex, challenging the conventional assumption of Mg^2+^ ion formation. This insight provides an intuitive explanation for the postulated presence of (Coulombically) univalent Mg^+^ ions, and the absence of protective oxide/hydroxide layers normally formed under anodic/oxidizing conditions. The discovery of this unexpected and unconventional reaction mechanism is crucial for identifying new strategies for corrosion prevention and can be transferred to other metals.

Controlling material degradation in chemically harsh environments is an outstanding challenge for future sustainable technologies. Examples are electrochemical energy conversion and storage solutions,^1−6^ green metallurgy,^7,8^ and lightweight structural materials.^9,10^ The need to understand the fundamental corrosion mechanisms in such environments is highlighted by a number of deceptively simple, yet poorly understood degradation reactions such as the anomalous dissolution of metals under anodic conditions; their precise mechanistic details have remained elusive since their discovery more than 150 years ago.^11^

Magnesium is a prototypical example.^12^ Mg alloys are attractive materials for mechanical engineering or batteries,^13^ due to their lightweight, high-abundance, and low environmental impact. However, with Mg being one of the most reactive metals, a major technical weakness is its corrosion when it comes into contact with water. Many of the properties of magnesium in water are puzzling and not understood. For example, under anodic polarization, where magnesium dissolves, it simultaneously shows extreme rates of hydrogen evolution (HE), which would normally be expected exclusively for cathodic potentials. According to the Butler–Volmer equation, HE should decrease exponentially when increasing the potential toward the anodic direction. In marked contrast, however, magnesium and its alloys feature strongly enhanced HE with increasing anodic polarization. This effect is referred to in the literature as the “negative difference effect” or “anomalous hydrogen evolution reaction”.^10,11,14^ Multiple models have been proposed^15,16^ to explain the origin of the anomalous hydrogen evolution. The “enhanced catalytic activity mechanism”^17^ suggested the enrichment of impurities more noble than Mg or the formation of local active sites to catalyze the HE on the anode. However, the specific nature of the catalyst that drives the anodic HE has remained unknown.^17,18^

Even more puzzling, the amount of Mg dissolved was observed to be greater than coulometrically expected, assuming that Mg was oxidized to the dipositive Mg^2+^ ion,^19^ cf. Figure 1a. These findings were taken as evidence for the existence of a “unipositive Mg^+^ ion mechanism”.^20^ In a series of follow-up experiments, it was demonstrated that the unipositive Mg ions have a lifetime of several minutes in aqueous solutions and are able to reduce other species even macroscopic distances away from the oxidizing Mg anode.^19,21^ On the other hand, the unipositive Mg^+^ mechanism has been challenged on the grounds that such an ion should be extremely short-lived. So far, there is only indirect evidence for the existence of Mg^+^.^10^ Atomic emission spectroelectrochemical experiments,^22,23^ which clearly distinguish different oxidation states, found direct evidence only for the divalent Mg^2+^. Yet, this anomalous dissolution behavior is not limited to Mg, but has been observed, e.g., for Fe, Cr, and Zn as well.^24^ Despite their fundamental importance, however, the existence and exact chemical nature of the postulated unipositive metal ions as well as the precise atomistic reaction mechanisms responsible for anomalous dissolution have remained elusive.

**Figure 1:**
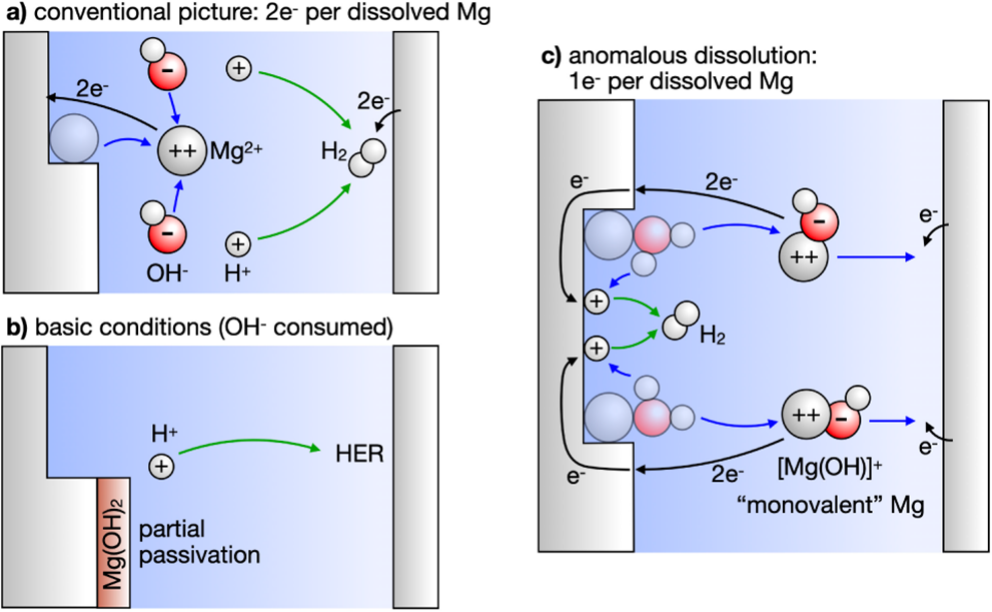
(a) Conventional picture of Mg corrosion: dissolution proceeds via formation of divalent Mg^2+^ ions, resulting in the transfer of 2e^–^ per dissolved Mg ion. In solution, Mg^2+^ reacts with OH^–^ and precipitates. (b) Under basic conditions, OH^–^ may be consumed, forming a hydroxide layer that partially passivates the Mg surface. (c) In the present work, we reveal a low barrier pathway where Mg dissolves as an effectively monovalent [Mg^2+^(OH)^−^]^+^ ion complex.

First-principles techniques could be the method of choice to reveal the origin of these anomalous dissolution reactions. However, studies that explore the corrosion process taking into account the full complexity of the realistic surface water interface are still lacking.

Via *ab initio* thermopotentiostat molecular dynamics simulations, we demonstrate that the hypothesized unipositive metal ions are in fact ion complexes, consisting of a divalent metal ion and an OH^–^ group. In the conventional picture, cf. Figure 1a, Mg dissolves via the formation of divalent Mg^2+^ ions, transferring 2e^–^ to the metal surface per dissolved Mg ion. Under basic conditions, cf. Figure 1b, water adsorbs dissociatively, partially passivating the metal surface via formation of a Mg(OH)_2_ surface hydroxide. In the present work, we identified an energetically and kinetically favorable reaction pathway where a surface metal atom becomes solvated in conjunction with an attached surface hydroxyl group (Figure 1c). This pathway completely circumvents the passivating nature of the hydroxide film. In turn, this reaction process supports a continued dissociative water adsorption, explaining the anomalous hydrogen evolution at the anodically polarized surface. The effectively unipositive [Mg^2+^(OH)^−^]^+^ ion complex is responsible for the observed amount of metal dissolved being larger than coulometrically expected. The [Mg^2+^(OH)^−^]^+^ ion complex may subsequently decay into the divalent metal ion via the reduction of another species. We expect it to be long-lived due to a strong Coulomb barrier separating the effectively unipositive ion complex from prospective reactants such as H^+^.

To reveal the dissolution mechanism and the nature of the unipositive Mg^+^ ion, we described the solvated Mg-surface by a supercell containing a 6-layer slab oriented in the (1 2 3̅ 15) direction and 64 explicit water molecules. Dissolution is generally understood to proceed via kink atoms due to their weaker bonds and the greater exposure of kink-sites to both adsorbing molecules and the electric field. We therefore induced a miscut in the Mg-slab, resulting in a surface with two kink-sites in the supercell. Already during equilibration under open-circuit conditions, a water molecule adsorbs dissociatively at one of the kink-sites according to
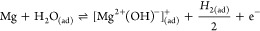
1Two further H_2_O adsorbed subsequently at the same kink-site as intact molecules, leading to the configuration shown schematically in Figure 2i. For clarity, only the participating water molecules are shown. Under open-circuit conditions, this configuration remained stable on the time scale of our simulations.

**Figure 2:**
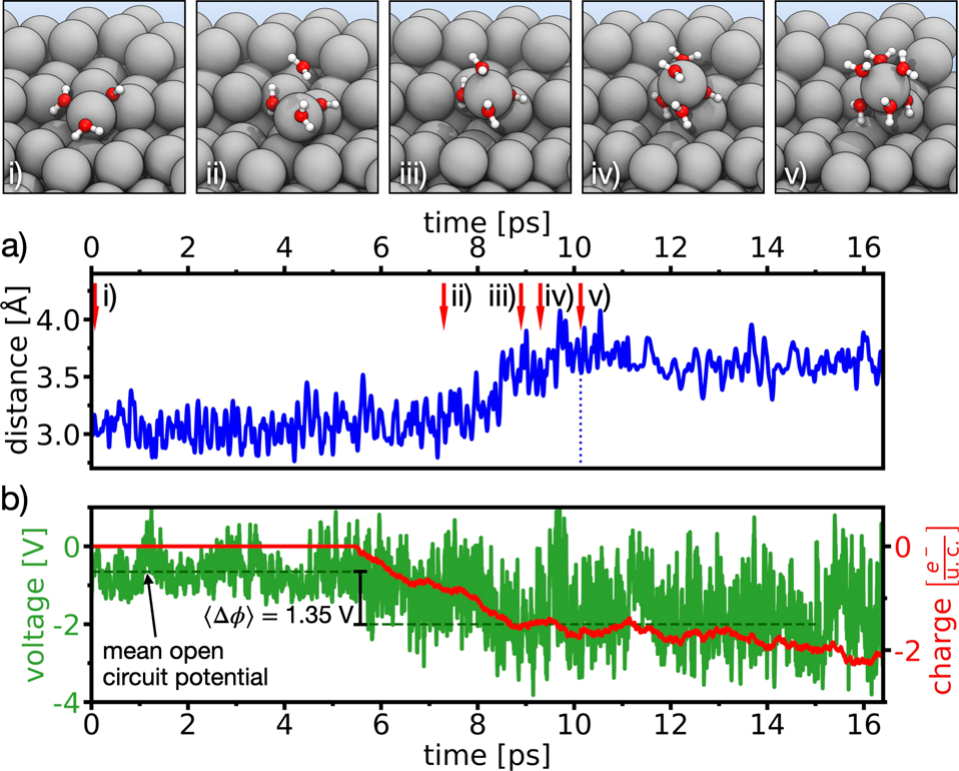
Solvation shell formation at kink-site Mg: (a) normal distance between the dissolving kink-site Mg-atom and its Mg bonding partner located directly underneath. The top panel shows schematic representations of the structural evolution along the AIMD trajectory, at times marked by red arrows. For clarity, only water molecules belonging to the solvation shell of the Mg^2+^ ion are shown explicitly. (b) Local system potential (green curve) and counter charge (red curve) used to balance the surface charge on the working electrode. Until turning on potential control at time *t* = 5.5 ps, the green dashed line marks the mean open circuit potential of ⟨ϕ(*t*)⟩ = −0.65 V, obtained in our simulations. Afterward the green dashed line indicates the targeted potential of ⟨ϕ(*t*)⟩ = −2 V. The Mg^2+^ ion remains attached to the surface via a hydroxyl bridge, see text.

In order to drive a dissolution reaction, we subsequently polarized the Mg slab anodically. The electrode charge is controlled by our recently introduced thermopotentiostat.^25,26^ Within 1.6 ps after switching on the thermopotentiostat with a target potential of ⟨ϕ⟩ = −2 V, a fourth water molecule approached the kink atom (cf. Figure 2ii) and adsorbed (Figure 2iii), starting to form a solvation shell. The kink-atom is then increasingly being lifted out of the surface. Figure 2a shows the distance parallel to the surface normal between the kink-atom and its Mg bonding partner underneath. A maximum extension of 3.6 Å is reached after the solvation shell is completed (Figure 2iv,v). Although the surface is charged with 2 additional electrons (cf. Figure 2b), indicating that the kink atom is now fully ionized, the solvated Mg^2+^ ion remains firmly bound to the surface: in conjunction with the hydroxyl group created in the reaction (eq 1), the solvated ion forms an [Mg^2+^(OH)^–^]_ad_^+^ complex, where the hydroxyl group connects the kink atom to its nearest Mg neighbor (cf. Figure 2v). We speculate that this process is common to anodically polarized metal surfaces. A “place-exchange mechanism”, where a surface-adsorbed oxygen and a metal atom underneath exchange their position, was first proposed by Lanyon et al.,^27^ later observed by Vetter and Schultze^28^ for Pt surfaces and reexamined more recently by Rost et al.^29^

This hydroxyl bridge bond is highly stable. Removing any water molecules except the ones constituting the solvation shell and separating the [Mg^2+^(OH)^–^]_ad_^+^ ion complex from the surface in a vacuum calculation, we estimated the binding energy to be ∼2 eV. Such a large binding energy is inconsistent with the experimentally observed high dissolution rates.^10,11,14^ In order for the dissolution to proceed, we therefore expect the breaking of the hydroxyl bridge bond to be catalyzed by its surrounding environment. Indeed, our simulations showed a possible candidate: a concerted double proton transfer from a neighboring adsorbed water molecule via a solvated H_2_O molecule that is hydrogen bridge bonded to the hydroxyl group (cf. Figure 3i) relocates the hydroxyl group laterally to a neighboring site. Thereby, the [Mg^2+^OH^–^]^+^ ion complex is oxidized to Mg^2+^ and left with a complete solvation shell consisting of 6 H_2_O molecules (Figure 3ii)
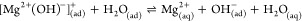
2As a result, the Mg^2+^ ion quickly moved into the liquid water region (dashed blue line in Figure 2), leaving the hydroxyl group behind on the surface.

**Figure 3:**
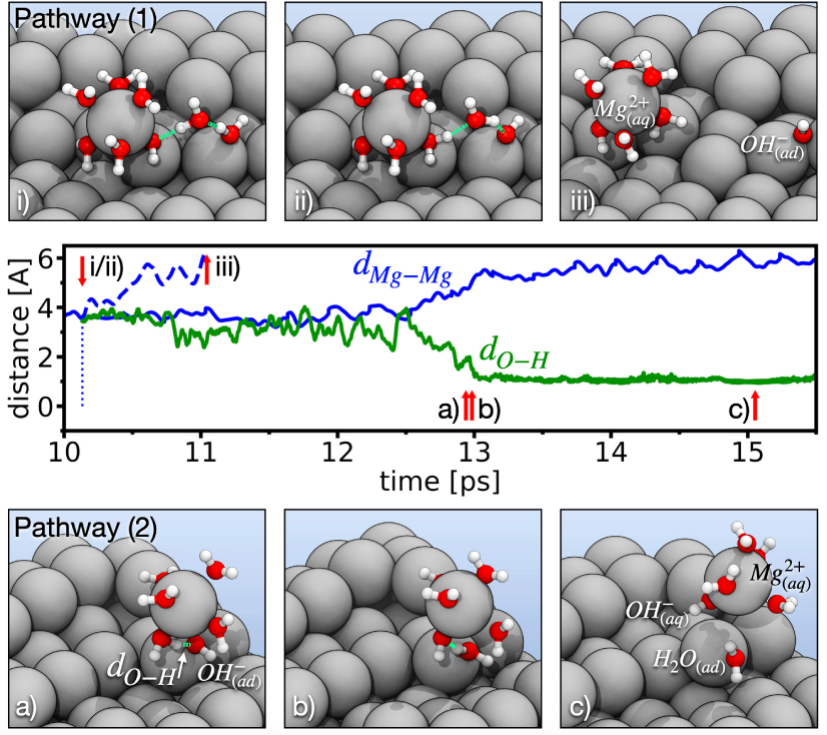
Two distinct pathways for Mg-dissolution. (1) A concerted double proton transfer from a surface adsorbed H_2_O molecule to the hydroxyl bridge releases the Mg_(ad)_^2+^ ion into solution. The corresponding normal distance between the dissolving kink-site Mg-atom and its Mg bonding partner underneath is indicated by the blue dashed line. The hydroxyl group remained on the surface. (2) Alternatively, an intra solvation shell single proton transfer to the hydroxyl bridge equally detaches the Mg_(ad)_^2+^ ion from the surface (solid blue line). The green line denotes the distance between the transferring proton and the hydroxyl bridge. The hydroxyl remains attached to the Mg_(aq)_^2+^ ion, resulting in an effectively +1 charged [Mg^2+^(OH)^–^]_(aq)_^+^ ion complex.

This double proton transfer proceeding on the surface is, however, not the only conceivable reaction to catalyze the dissolution. In order to search for alternative processes, we moved the Mg_(ad)_^2+^ ion with a constant velocity of *v* = 2/3 Å/ps parallel to the surface normal into the solution. In response, one of the H_2_O molecules forming the solvation shell turned one of its OH-bonds toward the hydroxyl group. In Figure 3, we show the distance between the hydrogen in the corresponding OH-bond and the oxygen atom of the hydroxyl group (green solid curve). In the time frame from 10.8 to 12.5 ps, multiple transfer attempts are visible, until at ∼13 ps an intra solvation shell single proton transfer occurs to the hydroxyl group (Figure 3a,b). Thereby, the [Mg^2+^(OH)^–^]_(ad)_^+^ ion complex as a whole becomes fully solvated and moves into the liquid water region (solid blue curve, Figure 3c)
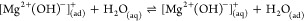
3We emphasize that the outcome of these two competing processes is fundamentally different. For reaction (eq 2), the hydroxyl remains on the surface. Therefore, the surface will be quickly hydroxylated and becomes electrochemically passive. No more dissociative H_2_O adsorption is possible, preventing any further anomalous hydrogen evolution. Reaction (eq 3), on the other hand, removes the hydroxyl group from the surface of the metal into the solution, leaving the next kink site exposed to further dissociative H_2_O adsorption. It is therefore only the reaction (eq 3) that is associated with ongoing anomalous hydrogen evolution. Consistent with the experimental observation that only the unipositive Mg ion is associated with the anomalous anodic hydrogen evolution,^19,20^ the [Mg^2+^(OH)^–^]_(aq)_^+^ ion complex created in reaction (eq 3) is effectively charged +1. We therefore propose that the elusive unipositive Mg^+^ ion is in fact an [Mg^2+^(OH)^–^]^+^ ion complex. This interpretation is supported by the fact that the reaction Mg^2+^(OH)^−^ ⇌ Mg^2+^ + OH^–^ is known to have a p*K*_b_ value of 2.56.^30^ Hence, for pH > 11.44 the [Mg^2+^(OH)^–^]_(aq)_^+^ ion complex becomes the dominant species. Due to the hydroxylation of the surface, we speculate that the local pH becomes sufficiently large so that the ion complex may even be thermodynamically stabilized.

Moreover, refs (19) and (21) pointed out that the unipositive Mg^+^ ion remains stable for several minutes in aqueous solutions and is able to reduce other species even at macroscopic distances away from the Mg anode. Since the  ion complex is positively charged and requires, e.g., an H^+^_(aq)_ ion to oxidize to , we expect the complex to be able to reduce other species. In addition, due to the Coulomb barrier between  and , which both are positively charged, the ion complex will be rather long-lived. An alternative reaction to obtain  is the dissociation of the  ion complex into its constituents  and . Analogous to the reduction via other species, this reaction is kinetically hindered by the large Coulomb attraction between the positive  and the negative .

Next to applying a potential of *Ũ* = −1.35 V, we also performed simulations at weak anodic conditions (*Ũ* = −0.35 V), as well as cathodic conditions (*Ũ* = +1.15 V). *Ũ* corresponds to the voltage measured against open circuit conditions.

The reaction steps observed in our thermopotentiostat AIMD simulations imply the following model for Mg dissolution and the anomalous HER via unipositive Mg: starting from the hydroxylated surface (Figure 4a), two distinct reaction pathways are available. On the one hand, with a rate of (1 – *k*) the hydroxylated kink atom is first ionized as
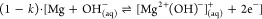
4and subsequently solvated according to (Figure 4c)
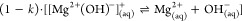
5

**Figure 4:**
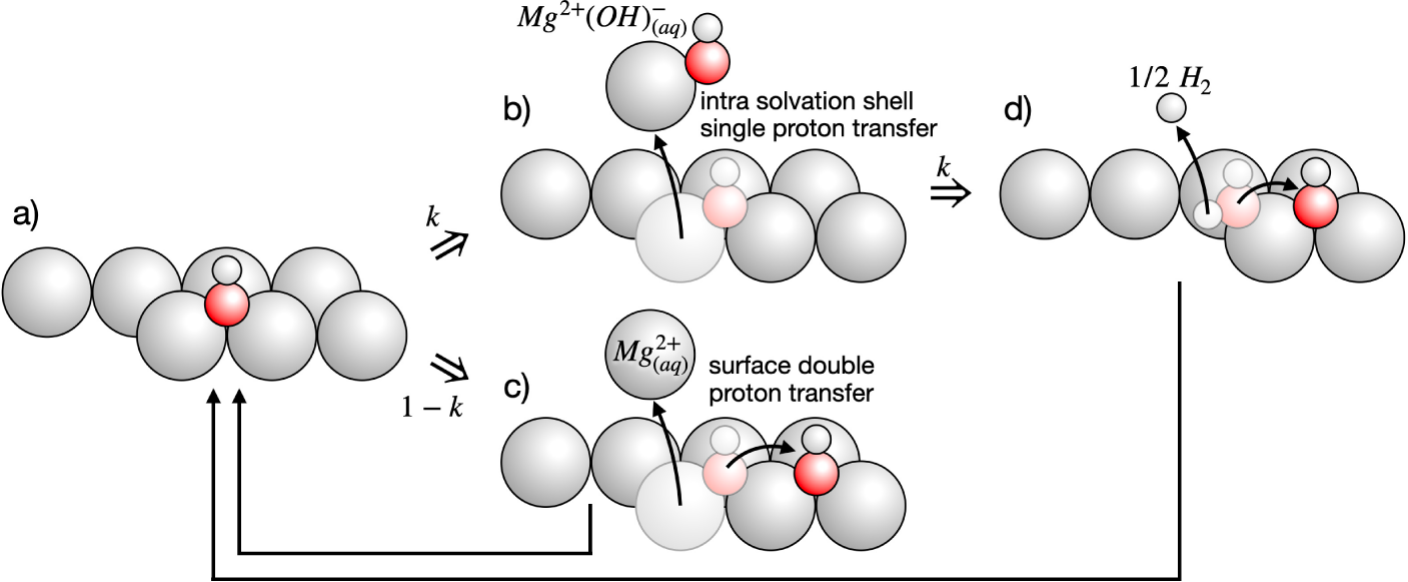
Reaction model for Mg dissolution and anomalous hydrogen evolution via unipositive Mg; see text.

After that, the process can start over at the next exposed kink atom (see Figure 4a). We note that summing eqs 4 and 5 results in
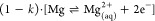
6

On the other hand, with a rate of *k* the  ion complex is solvated as a whole (Figure 4b). Since the hydroxyl group becomes detached from the surface, the surface is thereby left exposed to another dissociative adsorption event (Figure 4d)
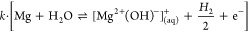
7This is the step that triggers the anomalous anodic hydrogen evolution reaction (HER), explaining why only the effectively unipositive Mg ion complex contributes to the anodic HER.

Eventually, the  complexes in the anolyte reduce other species—such as,  —and oxidize in the process according to
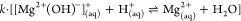
8Summing eqs 7 and 8 yields
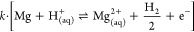
9We now see that eqs 6 and 9 are balanced by the cathodic half-reaction
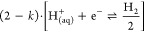
10so that the sum of eqs 6, 9, and 10 yields the well-known total balance equation
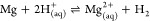
11We emphasize, that the key steps eqs 4, 5, and 7 are directly observable in our AIMD simulations. Only steps eqs 8 and 10 have been inferred.

The proposed model allows us to explain experimental observations^15^ showing that the amount of hydrogen produced per unit charge depends on the current density. We speculate that at higher current densities an increasingly larger fraction of the current will be carried by bivalent  ions, as opposed to the effectively monovalent ion complexes. As only the ion complexes contribute to the anodic HER, the amount of H_2_ produced per unit charge also decreases. Since the ion complexes efficiently remove hydroxyl groups from the surface while  does not, we expect the formation of distinctly different surface structures, depending on which pathway dominates.

Further approaches to test the proposed reaction mechanism include detection of the ion complexes themselves. We propose to extend the experiments by Petty et al.^19^ where fresh electrolyte flowing past an anodically polarized magnesium electrode was collected in another vessel. Hydroxide precipitation, induced, e.g., via titration by NaOH or KOH, may be able to determine the ratio between  and  present in the electrolyte. Alternatively, THz solvation shell spectroscopy, UV–vis and ^1^H or ^17^O NMR may be sensitive to the presence of  via changes in the  signals due to complexation.

In summary, by using *ab initio* molecular dynamics simulations of aqueous magnesium interfaces under potential control, taking into account the full complexity of the realistic metal–water interface, we have discovered a novel and completely unexpected reaction pathway. The identified dissolution product——naturally explains one of the most studied and debated corrosion mechanisms—the anomalous anodic hydrogen evolution, which has puzzled scientists since it was first reported more than 150 years ago. Our results clearly show that water is not just a spectator but an active reactant. Under anodic conditions, water dissociatively adsorbs to form a surface hydroxide. Subsequently, the interfacial water provides a low-barrier pathway for proton transfer reactions that allow the surface hydroxide to dissolve via the formation of  ion complexes. This pathway bypasses the usual passivation effect of surface films and explains the unusually high anodic corrosion rates and the chemical nature of the hypothesized solvated  ions. The discovery of such an unexpected reaction pathway also demonstrates the level and potential that ab initio molecular dynamics simulations have reached, thanks to recent methodological advances in the description of electrochemical interfaces.
